# Supplementary material for: Perceived learning difficulty associates with depressive symptoms and substance use among students of higher educational institutions in North Western Ethiopia: A cross sectional study
Source: PLoS One. 2020 Nov 10;15(11):e0240914. doi: 10.1371/journal.pone.0240914 (PMC7654822; doi:10.1371/journal.pone.0240914)
Supplement: Attachment — Submitted filename: Response to Reviewers.docx [file pone.0240914.s002.docx]

**Responses to reviewer comments**

1. Please ensure that your manuscript meets PLOS ONE's style requirements, including those for file naming. The PLOS ONE style templates can be found at

https://journals.plos.org/plosone/s/file?id=wjVg/PLOSOne_formatting_sample_main_body.pdf and

https://journals.plos.org/plosone/s/file?id=ba62/PLOSOne_formatting_sample_title_authors_affiliations.pdf

**Response:** thank you sending the links. We have now modified the manuscript (title page, author affiliations, the body of the manuscript and the file names accordingly. Since Plos One has its own section for funding, conflict of interest and availability of data, we have now removed the declarations section which contained these elements.

2.  We note that you have indicated that data from this study are available upon request. PLOS only allows data to be available upon request if there are legal or ethical restrictions on sharing data publicly. For more information on unacceptable data access restrictions, please see http://journals.plos.org/plosone/s/data-availability#loc-unacceptable-data-access-restrictions.

In your revised cover letter, please address the following prompts:

a) If there are ethical or legal restrictions on sharing a de-identified data set, please explain them in detail (e.g., data contain potentially sensitive information, data are owned by a third-party organization, etc.) and who has imposed them (e.g., an ethics committee). Please also provide contact information for a data access committee, ethics committee, or other institutional body to which data requests may be sent.

b) If there are no restrictions, please upload the minimal anonymized data set necessary to replicate your study findings as either Supporting Information files or to a stable, public repository and provide us with the relevant URLs, DOIs, or accession numbers. For a list of acceptable repositories, please see http://journals.plos.org/plosone/s/data-availability#loc-recommended-repositories.

We will update your Data Availability statement on your behalf to reflect the information you provide.

**Response:** thank you. We have now uploaded the dataset as an additional file along with the revised manuscript.

3. Thank you for stating the following in the Acknowledgments Section of your manuscript:

"This research was funded by Debre Markos University. But, the views in this study were fully
that of the authors and were not that of the funder.
TB is also financially supported by the Africa Mental Health Research Initiative (AMARI) within the
DELTAS Africa Initiative [DEL-15-01] as a post-doctoral fellow. The DELTAS Africa Initiative is an
independent funding scheme of the African Academy of Sciences (AAS)’s Alliance for Accelerating
Excellence in Science in Africa (AESA) and supported by the New Partnership for Africa’s Development
Planning and Coordinating Agency (NEPAD Agency) with funding from the Wellcome Trust [DEL-15-
01] and the UK government. TB was also supported financially by Debre Markos University."

We note that you have provided funding information that is not currently declared in your Funding Statement. However, funding information should not appear in the Acknowledgments section or other areas of your manuscript. We will only publish funding information present in the Funding Statement section of the online submission form.

Please remove any funding-related text from the manuscript and let us know how you would like to update your Funding Statement. Currently, your Funding Statement reads as follows:

"The funders had no role in study design, data collection and analysis, decision to
publish, or preparation of the manuscript."

**Response:** thank you indeed. We have now noted that our funding section submitted during our online submission stated as "The funders had no role in study design, data collection and analysis, decision to
publish, or preparation of the manuscript." We will update the funding information during submission. The update of the funding section will be made as follows. "This research was funded by Debre Markos University. But, the views in this study were fully
that of the authors and were not that of the funder.
TB is also financially supported by the Africa Mental Health Research Initiative (AMARI) within the
DELTAS Africa Initiative [DEL-15-01] as a post-doctoral fellow. The DELTAS Africa Initiative is an
independent funding scheme of the African Academy of Sciences (AAS)’s Alliance for Accelerating
Excellence in Science in Africa (AESA) and supported by the New Partnership for Africa’s Development
Planning and Coordinating Agency (NEPAD Agency) with funding from the Wellcome Trust [DEL-15-
01] and the UK government."

4. Please ensure that you refer to Figure 1 in your text as, if accepted, production will need this reference to link the reader to the figure.

**Response:** thank you. We have no figure in this manuscript

5. Please include captions for your Supporting Information files at the end of your manuscript, and update any in-text citations to match accordingly. Please see our Supporting Information guidelines for more information: [http://journals.plos.org/plosone/s/supporting-information](about:blank).

**Response:**

[Note: HTML markup is below. Please do not edit.]

Reviewers' comments:

Reviewer's Responses to Questions

**Comments to the Author**

1. Is the manuscript technically sound, and do the data support the conclusions?

The manuscript must describe a technically sound piece of scientific research with data that supports the conclusions. Experiments must have been conducted rigorously, with appropriate controls, replication, and sample sizes. The conclusions must be drawn appropriately based on the data presented.

Reviewer #1: Partly

**Response:** we have now formatted the manuscript according the journal’s formatting guidelines.

2. Has the statistical analysis been performed appropriately and rigorously?

Reviewer #1: No

**Response**: thanks for the comments of the reviewers on statistical aspects that guided as to revisit the statistical analysis again. In the current version of the manuscript, we have made modifications in Table 2, 3 and 4. We have checked the figures in the descriptive Table 2 and 3 using statistical package which was done using hand calculator. We have also added the descriptive for lifetime prevalence of substances in Table 2.

3. Have the authors made all data underlying the findings in their manuscript fully available?

The [PLOS Data policy](http://www.plosone.org/static/policies.action#sharing) requires authors to make all data underlying the findings described in their manuscript fully available without restriction, with rare exception (please refer to the Data Availability Statement in the manuscript PDF file). The data should be provided as part of the manuscript or its supporting information, or deposited to a public repository. For example, in addition to summary statistics, the data points behind means, medians and variance measures should be available. If there are restrictions on publicly sharing data—e.g. participant privacy or use of data from a third party—those must be specified.

Reviewer #1: No

**Response: we will be uploading the data accordingly. Thank you.**

4. Is the manuscript presented in an intelligible fashion and written in standard English?

PLOS ONE does not copyedit accepted manuscripts, so the language in submitted articles must be clear, correct, and unambiguous. Any typographical or grammatical errors should be corrected at revision, so please note any specific errors here.

Reviewer #1: No

**Response:** We have now carefully, edited the manuscript for language edits.

5. Review Comments to the Author

Please use the space provided to explain your answers to the questions above. You may also include additional comments for the author, including concerns about dual publication, research ethics, or publication ethics. (Please upload your review as an attachment if it exceeds 20,000 characters)

Reviewer #1: The authors aimed to investigate the prevalence and risk factors of depressive symptoms and substance use among university students in North-Western Ethiopia. Focusing on depression and substance use is quite significant both from a clinical and public health perspective. However, several points should be critically considered.

[Major comments]
The authors assert the importance of clinical depression and substance abuse; however, this cross-sectional study assesses the level of depressive symptoms and substance use that compromise with a flaw, limiting the clinical relevance which needs to be considered.

**Response:** PHQ-9 is DSM based and very common tool in low income countries to assess depressive symptoms. It has been locally validated in Ethiopia with cut off of five and above indicating depressive symptoms. People with PHQ-9 score of five and above have functional impairment and above a cut off 10 or more is clinical level as suggested in validation studies. Indeed, we assume that the findings have clinical relevance taking the limitations into account. We have changed the substance use assessment scores in count data to reduce limitation of the substance use scale. Use of some of the substance like shisha and cannabis are very rare and illegal in Ethiopia. We also focused only on the last three month data than the lifetime to focus on severity though we inserted the lifetime prevalence for interested readers.

Moreover, We preferred the cross sectional survey as a first step just to know the prevalence of the problem. We plan to conduct clinical based research once we knew the extent of the problem. Hopefully, we will conduct clinical and intervention based design to help adolescent university students who are depressed due to substance use.

The authors report that the prevalence of mild levels of depressive symptoms to be about 30%, and that of major depressive symptoms to be about 40%. However, the mean (SD) PHQ-9 score of the sample is not given, which makes it ambiguous whether the sample has marked depression symptoms that may cause noticeable problems in relationships with others and day-to-day school activities (functional impairment). The mean (SD) PHQ-9 score of the sample should be provided. The authors also report “mild” and “major” depressive symptoms based on PHQ-9; however, the score range of mild and major depressive symptoms category is not given.

**Response: thank you we have now provided the mean and standard deviation of the PHQ-9 score at the bottom row of in Table 1 while interpreting it in the text on page 11 of the manuscript. The cut off score was reported in the abstract section of the manuscript. in the current version, we have also included in the main body under “assessment” section (page 9).*-------**

The authors also report that substance use was assessed by a composite scale the asking the lifetime and the three months experience of using five substances: khat, alcohol, tobacco, shisha, and depressants, with an option of never (0), monthly (1), weekly (2) and daily (3). It appears that the scale does not evaluate noticeable problems (i.e., impairment) or distress that is an essential manifestation of substance use disorder; this assessment scale may show only recreational drinking or smoking. This makes it difficult to interpret the findings of this study whether it is referring recreational drinker or problematic alcohol users.

**Response:** We have changed the substance use assessment scores into count data to reduce limitation of the substance use scale. Use of some of the substance like shisa and cannabis are very rare and almost deviant behavior in Ethiopia. We also focused only on the last three month data than the lifetime to focus on severity though we inserted the lifetime prevalence for interested readers.

We have tried to check whether the respondents showed depressive symptoms or not due to substance use. Though not to the level of impairment, students reported that they used substances to escape from worries related to academic pressure , stress which may ultimately lead them to be dependent on these substance and unable to function without them.

[Minor comments]
1) In Table 2, the prevalence of males having at least mild depressive symptoms is shown as 70.5% (n=235). According to my calculation it is 49.5% (n/N=235/475 = 0.4947), please check.

**Response:** Check. Yes, the last column was calculated by hand from the remaining cells obtained from SPSS cross tabulation. So, that was typo error. Thank you


2) In Table 3, alcohol use in the last 3 months is shown as 173 (24.6%). The denominator should be all total substance users, 173/(28+25+173+33+24)=61.1%, please check.

**Response: the figure is referring to the prevalence and the denominator is the sample size. We have now made modifications and checked the figures for table 3.**

3) English editing is needed.

**Response: we have made careful language edits to the manuscript**

6. PLOS authors have the option to publish the peer review history of their article ([what does this mean?](https://journals.plos.org/plosone/s/editorial-and-peer-review-process#loc-peer-review-history)). If published, this will include your full peer review and any attached files.


If you choose “no”, your identity will remain anonymous but your review may still be made public.


**Do you want your identity to be public for this peer review?** For information about this choice, including consent withdrawal, please see our [Privacy Policy](https://www.plos.org/privacy-policy).

Reviewer #1: No

[NOTE: If reviewer comments were submitted as an attachment file, they will be attached to this email and accessible via the submission site. Please log into your account, locate the manuscript record, and check for the action link "View Attachments". If this link does not appear, there are no attachment files.]

While revising your submission, please upload your figure files to the Preflight Analysis and Conversion Engine (PACE) digital diagnostic tool, <https://pacev2.apexcovantage.com/>. PACE helps ensure that figures meet PLOS requirements. To use PACE, you must first register as a user. Registration is free. Then, login and navigate to the UPLOAD tab, where you will find detailed instructions on how to use the tool. If you encounter any issues or have any questions when using PACE, please email PLOS at [figures@plos.org](mailto:figures@plos.org). Please note that Supporting Information files do not need this step.

*In compliance with data protection regulations, you may request that we remove your personal registration details at any time.* [*(Remove my information/details)*](https://www.editorialmanager.com/pone/login.asp?a=r)*. Please contact the publication office if you have any questions.*
